# Phellem Cell-Wall Components Are Discriminants of Cork Quality in *Quercus suber*

**DOI:** 10.3389/fpls.2019.00944

**Published:** 2019-07-30

**Authors:** Carla Pinheiro, Stefanie Wienkoop, João Feio de Almeida, Cecilia Brunetti, Olfa Zarrouk, Sébastien Planchon, Antonella Gori, Massimiliano Tattini, Cândido Pinto Ricardo, Jenny Renaut, Rita Teresa Teixeira

**Affiliations:** ^1^Faculdade de Ciências e Tecnologia, Universidade NOVA de Lisboa, Lisbon, Portugal; ^2^Instituto de Tecnologia Química e Biológica, Universidade NOVA de Lisboa, Lisbon, Portugal; ^3^Department of Ecogenomics and Systems Biology, University of Vienna, Vienna, Austria; ^4^UCIBIO – REQUIMTE, Faculdade de Ciências e Tecnologia, Universidade NOVA de Lisboa, Caparica, Portugal; ^5^National Research Council of Italy, Trees and Timber Institute, Florence, Italy; ^6^Department of Agri-Food Production and Environmental Sciences, University of Florence, Florence, Italy; ^7^Luxembourg Institute of Science and Technology, Belvaux, Luxembourg; ^8^Institute for Sustainable Plant Protection, National Research Council of Italy, Florence, Italy; ^9^Instituto Superior de Agronomia, Universidade de Lisboa, Lisbon, Portugal

**Keywords:** hydrolysable tannins, proteomics, targeted metabolomics, soluble phenolics, cell-wall immobilized phenolics

## Abstract

Cork is a renewable, non-wood high valued forest product, with relevant ecological and economic impact in the Mediterranean-type ecosystems. Currently, cork is ranked according to its commercial quality. The most valuable planks are chosen for cork stoppers production. Cork planks with adequate thickness and porosity are classified as stoppable quality cork (SQC). The chemical composition of cork is known, but the regulation of metabolic pathways responsible of cork production and composition, hence of cork quality, is largely unknown. Here, we tested the hypothesis that post-genomic events may be responsible for the development of SQC and N-SQC (non-stoppable quality cork). Here, we show that combined proteomics and targeted metabolomics (namely soluble and cell wall bound phenolics) analyzed on recently formed phellem allows discriminate cork planks of different quality. Phellem cells of SQC and N-SQC displayed different reducing capacity, with consequential impact on both enzymatic pathways (e.g., glycolysis) and other cellular functions, including cell wall assembly and suberization. Glycolysis and respiration related proteins were abundant in both cork quality groups, whereas the level of several proteins associated to mitochondrial metabolism was higher in N-SQC. The soluble and cell wall-bound phenolics in recently formed phellem clearly discriminated SQC from N-SCQ. In our study, SQC was characterized by a high incorporation of aromatic components of the phenylpropanoid pathway in the cell wall, together with a lower content of hydrolysable tannins. Here, we propose that the level of hydrolysable tannins may represent a valuable diagnostic tool for screening recently formed phellem, and used as a proxy for the quality grade of cork plank produced by each tree.

## Graphical Abstract

The shikimate and the core phenylpropanoid pathways are involved in the synthesis of the polyphenols identified in cork producing cells. Stoppable cork quality producing cells are characterized by higher incorporation of aromatic components of the phenylpropanoid pathway in the cell wall, together with a lower content of hydrolysable tannins.

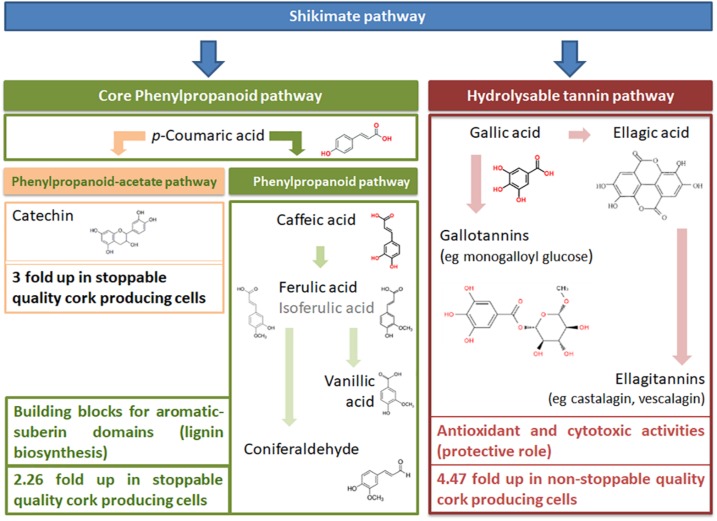

## Introduction

Cork is a renewable natural product, and its industrial exploitation allows maintain the *Quercus suber* forests, characteristic landscape of the Mediterranean ecosystem. These ecosystems are designated as “montado” or “dehesa” in Portugal or Spain, respectively. The single out characteristic of *Q. suber* is the continuous production of a protective cork layer from the phellogen, a secondary meristematic tissue. Chemically, cork consists of both insoluble and soluble components (Pereira, 2007). The insoluble components include aliphatic suberin (often designated as suberin), aromatic suberin (designated as lignin-like), cellulose and hemicelluloses (Silva et al., 2005; Pereira, 2007). Soluble components, or “extractives,” mostly consist of lipids and phenolics. There a is large variability in cork composition, since cork may contain 33–50% aliphatic suberin; 13–29% lignin-like aromatic suberin; 6–25% polysaccharides; 13–24% extractives, and 1–7% ash (Silva et al., 2005). The most abundant extractives are typically waxes and tannins (Silva et al., 2005).

This large variability in the chemical composition coupled with physical properties determines the suitability of cork for the transformation industry hence, its economic value (Lauw et al., 2018). Cork is commercialized as planks, i.e., the rectangular parcels of the outer bark that is separated from the tree along the phellogenic layer without damaging of this meristematic layer that holds the capacity of regeneration right after the debarking process. There is evidence that plank thickness and porosity are major discriminants of cork quality, since stoppable quality cork (SQC) planks ought to display 22–40 mm thickness with limited structural discontinuities (Pereira, 1998). Thicker planks show high cell number per annual ring growth and high cell prism height, i.e., these cells are larger (Silva et al., 2005). The thickness of the cork layer is largely dependent on both genetic and environmental factors, determinant for cell wall thickening development, allowing a more extensive cell expansion and promoting cell division.

There is evidence that the genetic regulation of meristem activity is mostly expressed during spring-summer, when the phellogen becomes more active (Silva et al., 2005; Boher, 2017). For instance, the expression levels of cyclins (promoters of cell cycle progression) were higher in 1-year cork branches of SQC producing trees, whereas in branches producing N-SQC cell expansion is constrained by the up-regulation of genes involved in primary cell wall strengthening (Teixeira et al., 2017). Cork planks, having an elevated number of wider cells, display sustained biosynthesis of cell-wall components, such as suberin-domains and polysaccharides, whose deposition requires biochemical regulation. Currently, the cell wall is described as a sensor, integrating the environmental cues in cell growth (Johnson et al., 2018). Consequently, its biomechanical properties will influence wall flexibility and strength. Recent reports point out to environmental influence on cork annual growth, porosity and thickness (Inácio et al., 2017; Lauw et al., 2018).

Previous transcriptome analysis did not provide clear discrimination between N-SQC and SQC differentiating cells (Teixeira et al., 2014, 2017). It is reasonable to hypothesize that mechanisms operating at post-transcriptional and post-translational levels may have been responsible for modified protein activity. Indeed, post-transcriptional control of phellogen activity via microRNAs has been previously reported (Chaves et al., 2014). Proteomics has the potential to evaluate differences and similarities between cork quality groups. Highly distinctive protein patterns will describe major biochemical differences between cork quality groups, while highly similar protein patterns will support the fine regulations of the phellogen metabolic activity.

Phenolic compounds display multiple roles in plants, including signaling, enzyme activity regulation and structural roles (Weisshaar and Jenkinst, 1998). They also perform antioxidant functions reducing the generation of reactive oxygen species (ROS) and the ROS content once they are formed. N-SQC producing cells were found to have higher soluble phenolics content than SQC: This was associated to stress responses and to the protective role of cork, a hydrophobic barrier against external constraints. It has been hypothesized that N-SQC producing trees may suffer from more severe stressful conditions compared to the SQC counterparts (Teixeira et al., 2014). Therefore, it is important to understand if a higher proportion of soluble phenolics in N-SQC cork producing cells is associated with a lower proportion of cell-wall phenolic compounds.

Our working hypothesis is that, N-SQC producing cells need to invest in soluble phenolics and the metabolic trade-off is to convey fewer building blocks for suberization. In addition, phenolic compounds have the potential to modulate the cellular oxidative-status, which can be relevant for phellogenic meristematic activity. Favored cell-proliferation and cell expansion over several growing years will have a positive impact on plank thickness, one of the cork quality traits evaluated and valued by the cork industry.

## Materials and Methods

### Sample Collection

Samples were taken from five trees producing cork of stoppable quality (SQC) and from five trees producing non-stoppable quality cork (N-SQC). The chosen cork oaks had an estimated age of 50–70 years and were located in southern Portugal. N-SQC was collected in the area of Coruche, Ribatejo (38°46′N, 8°39′W) and SQC at Serra do Caldeirão, Algarve (37°15′N, 7°59′W). Soil (Cardoso, 1965, 1974) and climatic data^1^ are summarized in Table 1. The classification of cork quality was based on visual observation, taking into account the criteria used by the industry: thickness and homogeneity, porosity and presence of defects (Pereira, 1998, 2007). SQC planks displayed a reduced number of discontinuities and a minimum thickness of 28 mm (before cooking). N-SQC displayed a maximum thickness of 17 mm (before cooking) and contained an elevated number of discontinuities that completely cross the plank.

**TABLE 1:** Location, climatic data, and soil characteristics for sampling locations.

	**N-SQC**	**SQC**
Sampling location	Coruche	Serra Caldeirão
Sampling location (GPS coordinates)	38°46′N, 08°39′W	37°15′N, 07°59′W
Altitude (m)	68	329
Metereological station location (GPS coordinates)	39°12′N, 08°44′W	37°01′N, 07°59′W
Mean air temperature (°C)^1^	17.0	17.9
Maximum temperature range (°C)^2^	14.7	10.1
Minimum temperature range (°C)^2^	8.5	7.2
*n*° months with negative temperature^2^	4	2
*n*° months with temperature > 35°C^2^	7	4
Mean year rainfall (mm)^1^	54.3	42.4
Soil classification^3^	Podzols	Lithosols

*^1^Climatic data refers to the 1981–2010 period (climatologic normal), available at www.ipma.pt/pt/oclima/normais.clima/1981–2010; ^2^Calcu- lated as the minimum (or maximum) monthly temperature range (1981–2010 period); ^3^(Cardoso, 1965, 1974).*

Samples were obtained by scratching the inner side of freshly removed planks comprising recently formed phellem with suberized (cork) and un-suberized cells (phelloid) and phellogen cells (meristem). Since cell division occurs during spring and summer, the cells in our samples are just a few weeks older. Altogether, samples represented distinct differentiation stages of cork (thereafter referred as to cork producing cells). Immediately after debarking, samples were collected and snapped frozen in liquid nitrogen. Samples were later on lyophilized, grinded using a ball mill and PTFE shaking flasks (Mikro-Dismembrator S, Sartorius) and stored at −80°C until further utilization.

### Total Phenolics Quantification

Following the protocol used by Zarrouk et al. (2012), 1 mL of acidified methanol (with 1% HCl) was added to 25 mg of sample (dry weight), and incubated at room temperature under gentle agitation in the dark, for the estimation of total soluble phenolics. Samples were extracted for 7, 24, 48, 72, 96, 168, or 216 h in order to optimize the extraction time. After centrifugation (18000 × *g* for 15 min at 4°C), supernatants were diluted 1/10 and used for total phenolics quantification, using the Folin Ciocalteau method modified for microplates (Singleton and Rossi, 1965; Zarrouk et al., 2012). Gallic acid was used as standard and the results were expressed in millimoles of gallic acid equivalents per gram of sample dry weight (mmol GAE g^–1^ DW).

The pellets resulting from 216 h extraction were further used for residues determination, which was achieved by adding 1 mL of acidified methanol (with 1% HCl) and incubating the tubes for additional 48 h. After centrifugation (18000 × *g* for 15 min at 4°C), the supernatants were diluted 1/10 and used for residues quantification as mentioned above.

### Soluble and Cell Wall Bound Phenolics Quantification by HPLC-DAD and HPLC-MS/MS

Freeze-dried samples (50 mg) were extracted with a methanol-water mixture 80/20 (v/v) pH 2.5 (with formic acid) at room temperature for 24 h under continuous stirring. The solution (containing soluble phenolics) was centrifuged and the supernatant collected. The pellet residues were used for extraction of wall bound phenolics.

The solution was dried under vacuum, the residue re-dissolved in water (pH 2.5), and then extracted three times with diethyl ether. Twenty microliters of the water fraction were injected into a PerkinElmer Flexar chromatograph equipped with a quaternary 200Q/410 pump and LC 200 diode array detector (all from PerkinElmer, Bradford, CT, United States). The diethyl ether fraction was dried and rinsed with MeOH/H_2_O (80/20) pH 2.5; then 40 μL were injected into the HPLC-DAD equipment. Pellet residues previously extracted for soluble phenolics were washed with ethanol and subjected to base hydrolysis, performed by adding 1 N NaOH at 80°C for 6 h, and then at room temperature for 18 h in test tubes. The supernatant was collected after centrifugation at 8000 × *g* for 10 min, acidified to pH 2 with 2 N HCl and then extracted three times with an equal volume of ethyl acetate (Blount et al., 2000). In addition, the pellet was washed two times with 3 mL of ethyl acetate. The organic phases were combined, taken to dryness and resuspended in MeOH/H_2_O pH 2.5 with HCOOH (8/2). Twenty microliters of this solution were injected into the HPLC-DAD equipment described above.

In the HPLC-DAD, compounds were separated on a 250 × 4.6 mm i.d (5 μm pore size) RP-C18 Zorbax SB kept at 30°C. Detection was carried out at 280 and 350 nm. Elution was performed using a linear gradient solvent system, at a flow rate of 1 mL min^–1^, consisting of H_2_O (A), CH_3_OH (B) and CH_3_CN (C), all containing 1% of HCOOH. The gradient profile was as follows: 0–2 min 98% A, 1% B, 1% C; 2–52 min from 1% of B and C to 49% of both and then returning to the initial conditions in 5 min. Quantification of individual polyphenols was performed using calibration curves (in 0.001 mg/mL to 0.2 mg/mL concentration range) of authentic standards (all from Extrasynthese, Lyon-Nord, Genay, France): vanillic acid, ferulic acid (ferulic acid, isoferulic acid), gallic acid (for galloyl HHDP glucose and gallotannins), p-coumaric acid for p-coumaric acid itself, caffeic acid (for caffeic acid derivatives), ellagic acid (for vascalagin/castalagin and ellagic acid itself) and epicatechin (for catechin) and expressed as mmol g^–1^ DW.

The identification of soluble phenolics of cork producing cells was performed through HPLC-MS-MS analysis (diagnostic fragments for each phenolic compound and the molecular ions are provided as Supplementary Table S1). Cell wall-bound polyphenols were assigned to the different classes (gallic acid, vanillic acid, ferulic, *p*-coumaric, and caffeic acid derivatives) using the comparison of retention time and UV-VIS spectra (Supplementary Table S1) with authentic standards and the data reported in literature (Fernandes et al., 2009; Harris and Trethewey, 2010; Ralph, 2010). Mass spectrometry analysis was conducted with an Agilent LC1200 chromatograph coupled with an Agilent 6410 triple quadrupole MS detector equipped with an ESI source (all from Agilent Technologies, Santa Clara, CA, United States). The column was a Poroshell SB-C18 (2.1 × 100 mm, 2.7 μm). The elution was performed with the same gradient reported above, at a flow rate of 0.3 mL min^–1^. Mass spectrometric detection was performed in the negative ion mode after electrospray ionization. The fragmentor was 180 eV. Sample volumes of 3 μL were injected. MS/MS spectra in the negative mode were obtained using argon as collision gas with the collision energy set at 5, 10, and 20 V.

Distribution of the several phenolic compounds detected through polyphenol classes was performed using the Phenol-Explorer database, v3.6 (Rothwell et al., 2013)^2^.

### Protein Electrophoresis and MS Analysis

Proteins were phenol extracted, precipitated with ammonium acetate and washed with acetone, in order to concentrate proteins and to remove contaminants that would interfere with the proteomic analysis. One gram of sample dry weight was used for protein extraction, following Isaacson et al. (2006) with minor modifications. Briefly, 10% (w/w) PVPP (polyvinylpolypyrrolidone) was added to the samples and 13.3 mL of extraction buffer was added (0.5 M Tris, pH 7.5; 0.7 M sucrose; 0.1 M KCl; 50 mM EDTA). After brief agitation, 13.3 mL Tris saturated phenol was added and incubated for 30 min on ice. Samples were centrifuged (15000 × *g*, 4°C, 30 min) and the upper phase was collected and saved. To the lower phase, an equal amount of extraction buffer was added and after 30 min on ice, samples were centrifuged (15000 × *g*, 4°C, 30 min). The upper phases were combined and precipitated overnight at −20°C with 5 volumes of 0.1 M ammonium acetate in methanol. After centrifugation (20000 × *g*, 30 min, 4°C), the supernatant was discarded and the pellet washed with 2 volumes of methanol, gently agitated for 1–2 min and centrifuged (20000 × *g*, 10 min, 4°C). The methanol washing was repeated and the pellet further cleaned with 2 volumes of acetone. After 1 h agitation at 4°C, samples were centrifuged (20000 × *g*, 10 min, 4°C). The acetone washes were repeated three more times and after the last centrifugation, the supernatant was discarded and the pellet dried under vacuum (1–2 h). Pellets were resuspended in 8 M urea and 4% CHAPS (w/v), gently agitated for 2 h, centrifuged (9500 × *g*, 15 min, 25°C) and the supernatant collected and desalted (PD miniTrap G25) against the same buffer. Protein was quantified with Bradford (Ramagli, 1999). One-hundred micrograms of protein were used for two-dimensional electrophoresis. Briefly, isoelectric focusing (IEF) was carried out with pH 3-10 immobilized strips (13 cm) and run to a cumulative 32120 Vh (Pinheiro et al., 2013). To improve the resolution, IEF was performed in the presence of 0.12% DeStreak Reagent (GE Healthcare). The second dimension SDS-PAGE was performed at 20°C with 12% resolving gels using the Hoefer SE 600 apparatus (GE Healthcare) at 10 mA per gel, for the first 15 min, and 20 mA per gel for the next 4 h, or until the bromophenol blue dye front had run off the gel. Precision Plus Protein All Blue Standards (Bio-Rad, Hercules, CA, United States) were used for molecular mass determinations. Gels were subsequently stained in Colloidal Coomassie Blue (Neuhoff et al., 1985) and images acquired with ImageScanner III (GE Healthcare). Image gel analysis was performed with Progenesis SameSpots 2D software v.4.5 (Non-linear Dynamics Ltd). The automatic analysis assigned 394 polypeptide spots, 355 spots being detected in at least four replicates of each cork quality group (n-1). These spots were submitted to MALDI-TOF/TOF for protein identification.

A Freedom EVO II workstation (Tecan) was used for the digestion. Briefly, gel plugs were washed twice with 50 mM ammonium bicarbonate solution in 50% v/v MeOH/MilliQ Water (Millipore) for 20 min and dehydrated twice for 20 min in 75% ACN. Proteins were digested with 8 μL of a solution containing 5 ng/μL trypsin (trypsin Gold, Promega) in 20 mM ammonium bicarbonate (overnight, 37°C). Digested peptides were extracted from the gel plugs with 50% v/v ACN containing 0.1% v/v TFA, dried and resolubilized in 0.7 μL of 50% v/v ACN/containing 0.1% v/v TFA. Peptides were spotted on a MALDI-TOF target and 0.7 μL of 7 mg/mL α-cyano-4-hydroxycinnamic acid in 50% v/v ACN containing 0.1% v/v TFA was added.

A MALDI mass spectrum was acquired using the Sciex 5800 TOF/TOF (Sciex). The 10 most abundant peaks, excluding known contaminants, were automatically selected and fragmented. MS and MS/MS were submitted to an in-house MASCOT server (version 2.3.1; Matrix Science^3^) for database-dependent identifications against the NCBInr database limited to the taxonomy *Quercus* (taxID3511; from February 01, 2019; 1,078,293 sequences). A second search was performed using the EST oak sequences from NCBI (taxID3511; from February 01, 2019; 870,474 sequences). Parameters were: peptide mass tolerance 100 ppm, fragment mass tolerance 0.5 Da, cysteine carbamidomethylation as fixed modification (alkylation was performed during the equilibration step between IEF and second dimension), and methionine oxidation, double oxidation of tryptophan, tryptophan to kynurenine and ethylation of glutamic acid as variable modifications. Kynurenine, resulting from tryptophan oxidation, is an artifact often observed during automatic digestion in our laboratory. Ethylation of glutamic acid is an artifact resulting from gel staining and destaining procedures. Up to two miscleavages were allowed. An identification was considered significant when at least two peptides passed the Mascot threshold score, or a single peptide passed twice this score. Manual checking of the spectrum allowed to confirm some identifications with slightly lower scores. When high-quality spectra were not matched to a protein, manual interpretation of the spectra was performed and/or the search parameters adjusted (semitryptic, single amino acid changes, post-translational modifications) to increase the sequence coverage of the identified protein. In our dataset, and regarding post-translational modifications, only acetylation was found, pointing out to a more technical than biological related modification. All identifications were validated manually. The mass spectrometry proteomics data have been deposited to the ProteomeXchange Consortium^4^ via the PRIDE partner repository (Vizcaíno et al., 2016) with the dataset identifier PXD014398.

For 45 protein spots, no MS spectra was obtained. Match with database sequences was found for 310 protein spots. Single, and significant, protein match was detected for 215 spots. For 69 polypeptide spots, and although at least one secure protein identification was obtained, the presence of other proteins was evident. Most of these protein spots (*n* = 66) displayed two matches. In several cases (11 spots), matches were achieved but no functional annotation was available. A bioinformatics analysis allowed retrieving the protein identity for these spots.

For each differentially expressed protein spot a manual annotation process was carried out. The peptide pool of each spot was scanned for uniqueness, and collinearity. Sequences sharing common sub-sequences were consolidated by concatenation prior to similarity search. The similar candidates were obtained through standard BLASTP (Boratyn et al., 2013) from amongst the plant entries in the NCBI Protein database (Agarwala et al., 2016). Only the top scoring hit in each comparison was retained for further analysis. This was true provided a reasonable balance between the degree of overall similarity and the significance (*e*-value) of the match. A flexible threshold policy was followed to accommodate the variation in length of the query peptides. Any existing InterPro (Mitchell et al., 2019) annotation in the target entries, and for the matched locations was transferred for the query. Whenever feasible, the pool of peptides obtained for each source protein was aligned to a known related sequence in order to sort them out along the original sequence. This was achieved using EMBOSS:needle (Rice et al., 2000) with BLOSUM35 substitution matrix, no end-gap penalties, and avoiding the insertion of indels in the alignment.

### Annotation of Peptides From the Whole Data Set

The whole set of spots, and their sequenced peptides was processed through a pipeline of automatic annotation that followed, roughly, the manual annotation already described. This process ensured that only relevant functional annotation was transferred to those peptides. All obtained peptide sequences were matched against UniProt entry sequences (The UniProt Consortium, 2019) using BLASTP (Boratyn et al., 2013). The matches were subsequently mapped on InterPro features already annotated on each of the matched UniProt entries. From the latter it was possible to transfer InterPro federated annotation like GO node references (The Gene Ontology Consortium, 2019), and EC activities (Nagano, 2005). The mapping of the UniProt matches onto their precise InterPro annotation pointed toward 524 corresponding entries in the latter database. These in turn, were associated to 111 Gene Ontology terms and 90 entries in the EzCatDB database^5^. The individual functional annotation of each peptide revealed at least 13 spots with more than one annotated function. The individual annotation of each peptide enabled us to avoid the common issue associated with the results of plain similarity searches – contamination by the annotation associated with unrelated parts of the subject sequence. After functional peptide annotation, they were clustered according to their parent protein spots.

Some classes of functional annotation were related to a wide scope of proteins, and the gel spots assigned to them are bound to lack in specificity for their role or localization in the studied cells. In order to be able to annotate them in a more specific subset of those functional annotation classes, a BLASTP (Boratyn et al., 2013) search against the NCBI Protein database (Agarwala et al., 2016) was conducted for some pertinent spots. The resulting matches were assessed for convergence on either activity or localization. Only relevant results were retained.

The location inside the cell was derived either from the InterPro GO annotation or from related protein similarity or phylogenetic analysis.

### Data Analysis

On the R platform (version 2.15.1) we used several packages for univariate analysis [Mann-Whitney *U*-test with Wilcox.test; Kolmogorov-Smirnov test with KS test; Student’s *t*-test with *t*-test (R Core Team, 2016)] and ade4TkGUI for principal component analysis (PCA) (Thioulouse and Dray, 2007).

In order to integratively analyze quantitative metabolite and protein data, a z-transformation was applied to multivariate statistics (PCA and Cluster heatmap-Pearson analyses), using the MATLAB (R2017a, the MathWorks, Inc., Natick, MA, United States.) package software tool COVAIN (v. 2019) (Sun and Weckwerth, 2012).

## Results

### Cork Quality Discrimination by the Soluble and Immobilized Phenolic’s of the Cork Producing Cells

**FIGURE 1:**
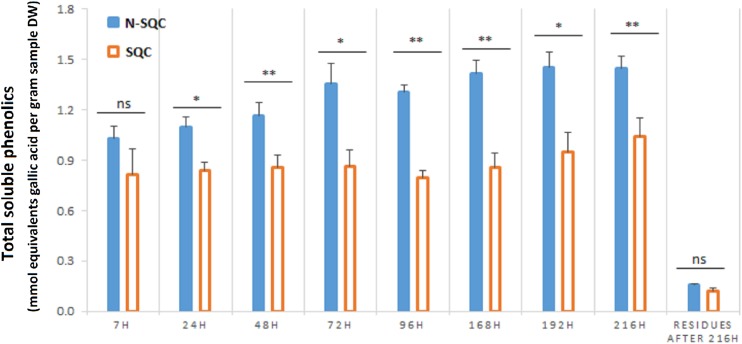
Folin-Ciocalteu based quantification of the total soluble phenolic compounds extracted from stoppable quality cork (SQC) and non-stoppable quality cork (N-SQC) producing cells. Extraction times between 7 h and 216 h were assayed. The remaining soluble phenolics after 216 h of extraction were also quantified. Data show the means ± standard error (*n* = 5). Significance levels between cork quality groups were assessed via Mann–Withney *U*-test (^*^p < 0.05; ^∗∗^*p* < 0.01).

Total phenolics, as estimated with the Folin-Ciocalteau method, were more abundant in N-SQC than in SQC, regardless the extraction time (Figure 1). However, this method typically overestimates soluble phenolics content, since it detects also a range of reducing compounds, such as ascorbate and reducing sugars (Georgé et al., 2005; Seruga et al., 2011). Therefore, the method is a better proxy for the reducing capacity of the sample (to which phenolics contribute), showing that N-SQC had a higher reducing capacity than SQC (around 40%).

Single phenolic compounds were quantified with HPLC-DAD. A higher content of soluble phenolic compounds was found in N-SQC producing cells (fourfold higher) than in SQC producing cells. As expected, HPLC-DAD analysis revealed a much lower content in total soluble phenolics compared to the Folin-Ciocalteau quantification method (it represents circa 3.3 and 1.2%, for N-SQC and SQC, respectively, Supplementary Figures S1A,B).

**FIGURE 2:**
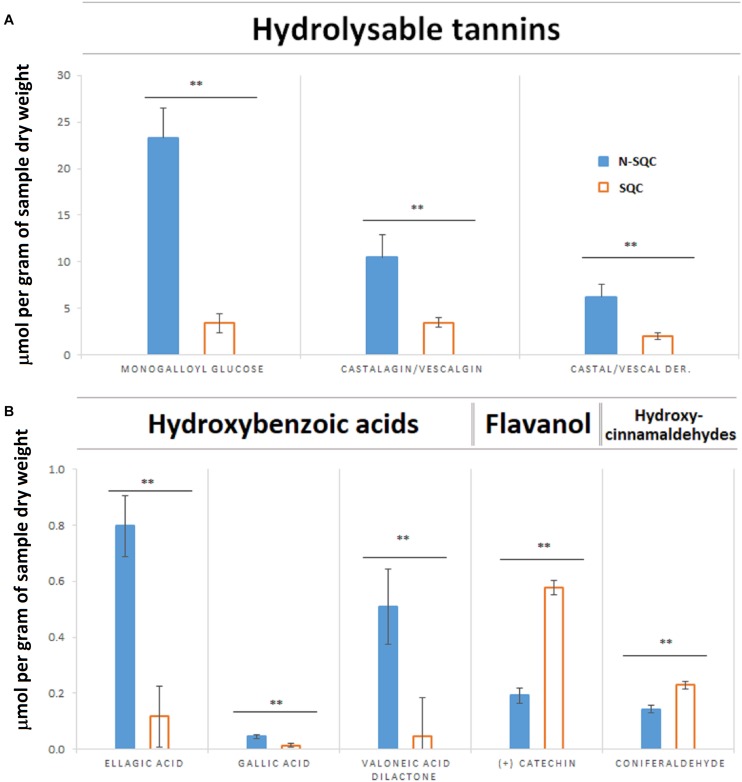
Quantification of soluble phenolic compounds extracted from stoppable quality cork (SQC) and non-stoppable quality cork (N-SQC) producing cells using HPLC-DAD. **(A)** Hydrolysable tannins. **(B)** Hydroxybenzoic acids belonging the class of phenolic acids (ellagic acid, gallic acid, and valoneic acid-dilactone), flavanols belonging to the flavonoids class (catechin) and hydroxycinnamaldehydes belonging to other polyphenols class (coniferaldehyde). Polyphenol classes and sub-class as described in the Phenol-Explorer database. Data show the means ± standard error (*n* = 5). Significance levels between cork quality groups were assessed via Mann–Withney *U*-test (^∗∗^*p* < 0.01).

The soluble phenolics fraction comprised two main phenolic classes: hydrolysable tannins and hydroxybenzoic acids. Hydrolysable tannins were the main component in both N-SQC (96%) and SQC (90%, Figure 2). However, hydrolysable tannins content was much higher in N-SQC than in SQC producing cells (40.03 vs. 8.95 mmol g^–1^ DW, respectively). N-SQC and SQC cells also differed for the main hydrolysable tannins: monogalloyl-glucose, the main component in N-SQC (around 56% of all phenolics detected) while in SQC, the isomers castalagin, vescalagin and their derivatives were the main compounds (∼70% of all phenolics detected). Hydroxybenzoic acids (ellagic acid, gallic acid, valoneic acid-dilactone), the second most represented class, were also more abundant in N-SQC than in SQC producing cells (1.35 mmol g^–1^ DW vs. 0.18 mmol g^–1^ DW, respectively). On the other hand, the amount of flavanol catechin and the hydroxy-cinnamyl-aldehyde coniferaldehyde in SQC producing cells was much higher (8.6%) when compared to N-SQC samples (0.8%). Catechin is the building block of condensed tannins, but in our dataset this class of phenolics was not detected.

**FIGURE 3:**
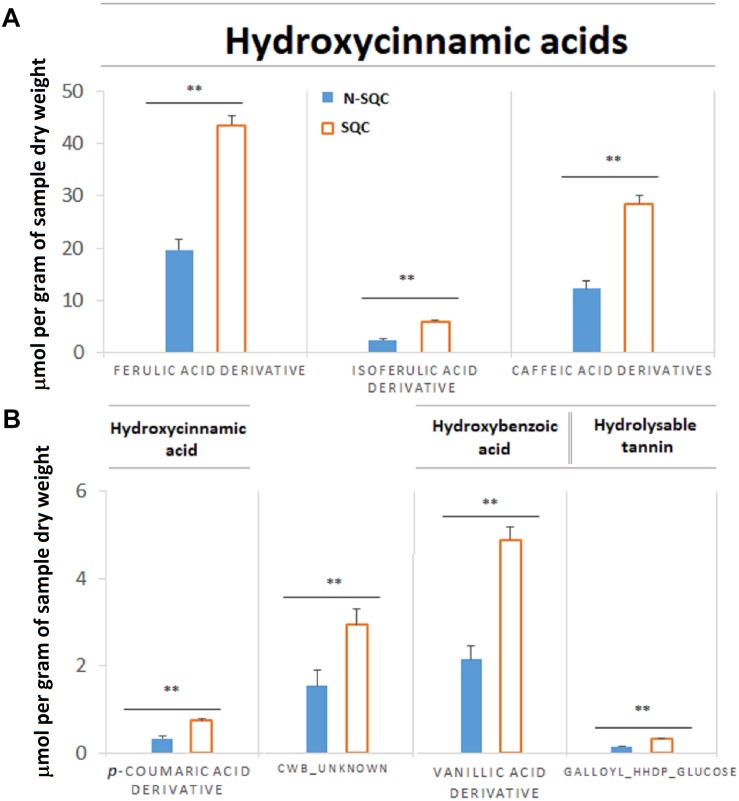
Quantification of immobilized phenolic compounds extracted from stoppable quality cork (SQC) and non-stoppable quality cork (N-SQC) producing cells after alkaline hydrolysis. **(A)** Major components are phenolic acids of the sub-class hydroxycinnamic acids (derivatives of ferulic acid, isoferulic acid, and caffeic acid). Six isoform candidates of caffeic acid were found and the quantification for each isoform is available as Supplementary Table S2. **(B)** Minor components are hydroxycinnamic acids (*p*-coumaric acid derivative) and hydroxybenzoic acids (vallinic acid derivative), hydrolysable tannin (galloyl-HHDP-glucose) and an unknown compound putatively assigned as a ferulic acid derivative (CWB_unknown). Polyphenol classes and sub-class as described in the Phenol-Explorer database. Data show the means ± standard error (*n* = 5). Significance levels between cork quality groups were assessed via Mann–Withney *U*-test (^∗∗^*p* < 0.01).

On the other hand, cell wall-bound phenolics were 2.25 times more abundant in SQC than in N-SQC producing cells (Figure 3), albeit the lack of differences between the type of phenolics detected. The cell wall bound phenolics consisted of 90% of hydroxycinnamic acids (ferulic, isoferulic, *p*-coumaric, and caffeic acid derivatives) and about 6% of hydroxybenzoic acids (vanillic acid derivatives). An unknown compound representing 4% of the cell wall-bound phenolics was also detected (Figure 3).

The PCA of the phenolic compounds (both soluble and cell wall-bound) allowed to clearly discriminate between SQC and N-SQC producing cells along the 1st component (77% of variation explained, Supplementary Figure S1C). Several metabolites involved in the phenylpropanoid pathway, including caffeic acid, ferulic acid and vanillic acid were positively related to SQC producing cells. In contrast, several metabolites of the hydrolysable tannin pathway were found positively related with N-SQC producing cells. The exceptions were catechin and coniferaldehyde that positively discriminated SQC.

### Cork Producing Proteome

Stoppable quality cork and N-SQC producing cells did not differ in the amount of solubilised protein (Supplementary Figure S2). The two-dimensional gel-based proteomics approach allowed the detection of 355 reproducible polypeptide spots, and these spots were found in both cork quality groups.

**FIGURE 4:**
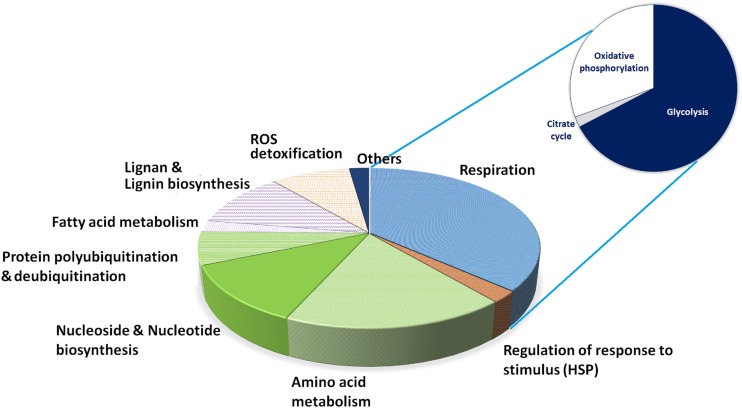
Relative distribution of proteins by reaction/process. The set of activities found for each spot after functional annotation was expertly assessed for relevant cellular reactions or processes, and here they are depicted by representation in terms of spots found. The glycolysis slice was further expanded for the sake of clarity on its individual component representation.

The high quality functional annotation of peptides described in the section “Materials and Methods” allowed us to retrieve the biological processes most represented in cork producing cells (Figure 4). These cells are characterized by possessing high glycolysis and energy production (37%) and amino acid metabolism, protein synthesis and turnover (39%), which is compatible with actively growing phases of the cell cycle. Considering the number of putative isoforms detected, the most represented enzymes involved in aerobic respiration were ATP synthase (EC 3.6.3.14), enolase (EC 4.2.1.11), fructose-bisphosphate aldolase (EC 4.1.2.13) and glyceraldehyde-3-phosphate dehydrogenase (EC 1.2.1.12). Enzymes involved in amino acid metabolism were detected, with a large preponderance of *S*-adenosylmethionine synthetase (EC 2.5.1.6) detected as 16 putative isoforms. Proteins involved in pyrimidine and purine nucleoside triphosphates biosynthesis, the activated precursors of DNA and RNA, were also largely represented (16 putative isoforms) along with proteins involved in protein turnover via the proteasome pathway (14 putative isoforms).

Fatty acid metabolism and lignin and lignan biosynthesis represented 13.4% of the annotated proteome, suggesting an active synthesis of secondary cell wall components. This percentage could be higher as six additional acyl-carriers proteins were identified (fatty acid metabolism) but the peptide annotation did not allow to securely add them to the proteome. Lignan biosynthesis was represented by proteins with NmrA-like domains (IPR008030) matching isoflavone reductase. Lignin biosynthesis was represented by caffeoyl-CoA *O*-methyltransferase, which belongs to the superfamily SAM-dependent *O*-methyltransferase (IPR029063). This enzyme is involved in the synthesis of feruloylated polysaccharides and in the formation of cell wall-bound ferulic acid polymers (i.e., secondary cell wall assembly and defense response). ROS metabolizing enzymes (catalase and peroxidases) represented 9% of the annotated proteome.

### Cork Quality Discrimination by the Proteome of Cork Producing Cells

**FIGURE 5:**
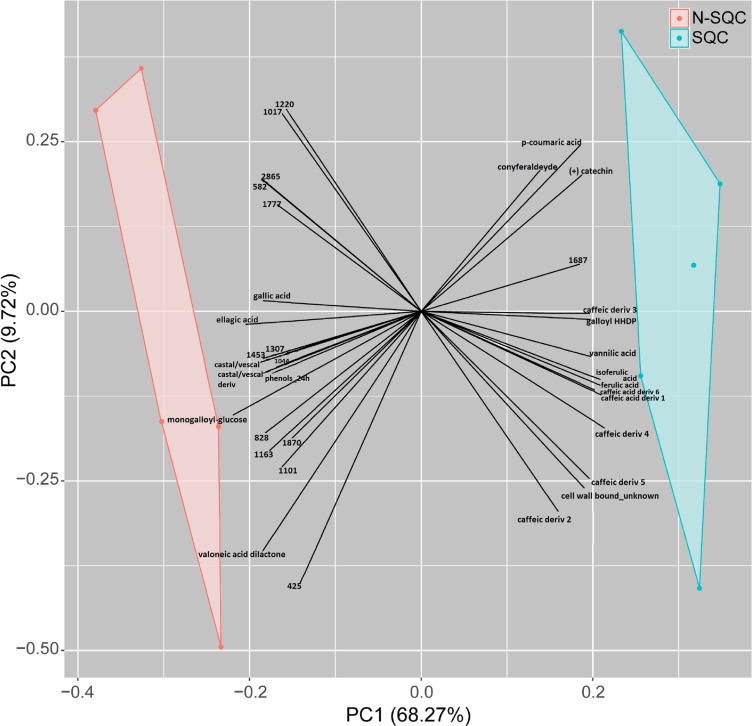
Integrative PCA of significantly different metabolite and protein abundance levels (ANOVA *p* < 0.05, *n* = 5, z-transform data, using COVAIN), distinguishing cork quality groups. Only significant variables are shown. All the phenolics were significantly associated with a cork quality group. The PCA loadings were used to build the plot (*x*- and *y*-axis).

**FIGURE 6:**
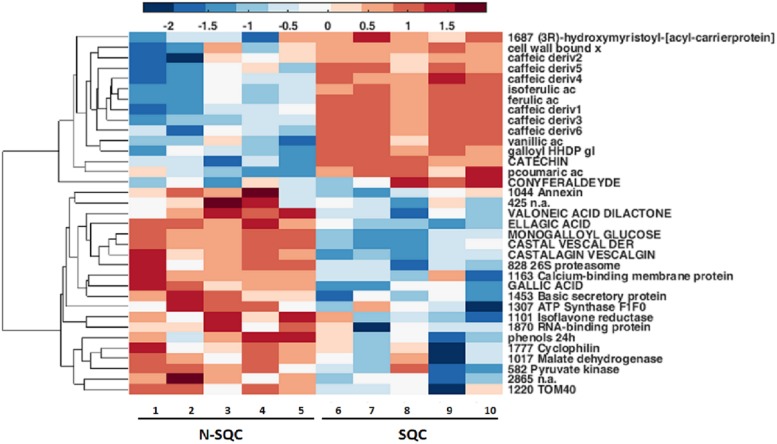
Integrative hierarchical cluster analysis of all statistically significantly changed metabolites and proteins (ANOVA, *p* < 0.05, *n* = 5, using COVAIN). Blue color means variables accumulated in N-SQC; red color indicates accumulation of compounds of SQC. All the phenolics were significantly associated with a cork quality group.

A PCA analysis of the proteome discriminated the cork quality groups (Supplementary Figure S2D) with 16% and 14% of the variation being explained along the 2^*nd*^ and 3^*rd*^ PCA axis. The integrative analysis of proteome and phenolic data was suitable to discriminate the examined cork quality groups (PC1 68%, Figures 5, 6). In detail, the groups largely differed for the phenolics component and less for the proteome component. Only 14 protein spots, a small fraction of the 355 spots detected (ANOVA statistics, Figures 5, 6 and Table 2) allowed discrimination. Unique protein identification was available for only 10 spots (Table 2 and Supplementary Table S3). Consistently, only a small fraction of the polypeptide spots differentially accumulated in cork quality groups (Supplementary Figure S2 and Supplementary Table S3). Similarly to previous reports (Pinheiro et al., 2013), the assessment of a given polypeptide spot as differently expressed accumulated was univariate test dependent. In the present work, we considered a gel spot signal to be distinct between cork quality groups when simultaneously assessed by the two non-parametric tests (*p* < 0.05 for MW; *p* < 0.08 for KS). In the proteome data set, 22 protein spots fulfilled this criterion with unique identification being achieved for 16 spots (Supplementary Table S3). This list largely overlapped with protein spots ascribed as discriminators between cork quality groups through the integrative proteomics and phenolic analysis (Table 2). Exceptions were verified for protein spots 425 (no protein identification achieved) and for 1777 (identified as cyclophilin). Altogether, our analysis identified 18 proteins as having quantitative differences between the quality groups, 14 of them having bigger volumes in N-SQC.

**TABLE 2:** Summary table of cork quality discriminant proteins following integrative data analysis (Inferno RDN tool) and peptide annotation.

**Spot volume (average ± st error)**				**Protein identification after peptide annotation**		
**Spot**	**N-SQC**	**SQC**	**Fold change (N-SQC/SQC)**		**Functional Annotation Transfer**	**Notes**
425	0.196 ± 0.014	0.158 ± 0.008	1.24		no information available^*^	
582	0.048 ± 0.002	0.034 ± 0.004	1.41	(i)	Pyruvate kinase	
				(ii)	Peptidase with M16 domain	
				(iii)	ATP synthase, F1 complex, alpha subunit	
828	0.104 ± 0.011	0.052 ± 0.004	2.00		26S proteasome regulatory subunit	
1017	0.067 ± 0.002	0.055 ± 0.004	1.22	(i)	Malate dehydrogenase, mitochondrial	
				(ii)	D-galacturonate reductase	
1044	0.666 ± 0.067	0.479 ± 0.033	1.39		Annexin	Organization of extracellular matrix; Signal transduction
1101	0.055 ± 0.007	0.032 ± 0.005	1.72		Isoflavone reductase	
1163	0.092 ± 0.003	0.066 ± 0.006	1.39		Calcium-binding membrane protein	Activation/inactivation of target proteins
1220	0.413 ± 0.023	0.295 ± 0.031	1.40		TOM40	Import of proteins into mitochondria
1307	0.283 ± 0.018	0.214 ± 0.019	1.32		ATP Synthase F1F0, mitochondrial	ATP synthesis on proton gradient
1453	0.126 ± 0.015	0.063 ± 0.008	2.00		Basic secretory protein (BSP)	Plant defense response; peptidase
1777	0.355 ± 0.026	0.263 ± 0.027	1.35		Cyclophilin	Protein folding
1870	0.616 ± 0.057	0.420 ± 0.048	1.47		RNA-binding protein, mitochondrial	Role in RNA transcription/processing during stress
2865	0.050 ± 0.004	0.036 ± 0.002	1.39		no information available^*^	
1687	0.030 ± 0.003	0.043 ± 0.002	0.70		(3R)-hydroxymyristoyl-[acyl-carrier-protein] dehydratase	Fatty acid biosynthesis; Biotin metabolism

With the exception of spots 425 and 1777, all the other protein spots were found differentially expressed between groups through univariate tests and when considering the proteome dataset alone. Detailed information is available as Supplementary Tables S3, S4. ^*^MS spectra not obtain for this spot.

One putative isoform of cyclophilin (spot 1777) was 35% more abundant in N-SQC. This was only one of four candidate cyclophilin isoforms and when all the presumed isoforms were considered, cyclophilin was found 53% more abundant in N-SQC producing cells.

Three isoforms candidates of isoflavone reductase were significantly more abundant in N-SQC, altogether representing 50% more volume when compared with SQC. Considering all the nine putative isoforms detected in our dataset, N-SQC producing cells exhibited 20% more volume of isoflavone reductase. These proteins were included in the lignan biosynthesis category due to its identification as isoflavone reductase that catalyzes the NADPH-dependent reduction of phenylcoumaran benzylic ethers. However, the detailed peptide analysis only confirms the identification of NmrA-like family domain containing proteins (IPR008030), described as negative regulators of the transcription factor AreA. NmrA-like proteins are a subgroup of the NAD(P)-binding domain (IPR016040) that overlaps with the NAD(P)-binding domain superfamily (IPR036291). Among the 33068 proteins that present the NmrA-like domain, 65 are from *Viridiplantae* – Arabidopsis and Oryza^6^. This superfamily includes many reductases (e.g., isoflavone reductase homolog P3 or pinoresinol -lariciresinol reductase 3 from Arabidopsis) and, in plants, this function seems to overlap with the role of negative regulator of transcription that is recognized in fungi.

One isoform of glutathione S-transferase dehydroascorbate reductase-like (spot 1354), was significantly more abundant in SQC (15 %). Considering all seven putative isoforms detected, no quantitative changes were found between the cork quality groups.

The protein detected in spot 1163 (40% more abundant in N-SQC) was identified as calcium-binding protein. Additionally, in three other spots, proteins were also identified as calcium-binding protein. These proteins share the EF-hand domain (IPR002048), which is detected in 473 and 344 proteins, respectively, in Arabidopsis and rice. The EF-hand motif is an evolutionary related calcium-binding domain exhibited by many calcium-binding proteins^7^. The biological role is diversified and the lack of additional information only permits to consider a potential calcium sensor role.

One putative isoform of annexin (spot 1044), a calcium-dependent phospholipid-binding protein, was differently abundant between the cork quality groups (40% more spot volume in N-SQC). When considering all three isoforms detected, N-SQC producing cells exhibit 30% more of this protein. Recent reports suggest multiple roles for annexin, including signal transduction, exocytosis and endocytosis and organization of the extracellular matrix^8^.

Spot 1870 was found to be differently accumulated in N-SQC (45%), the protein being identified as RNA-binding protein. This protein exhibits the RNA recognition motif domain (IPR000504), the same motif was detected within five other spots. Taking into consideration their similarity, three different proteins were most likely detected. Proteins of spots 1870, 1905, and 1928 were seen as potential isoforms and when considered together, they were significantly more abundant in N-SQC than in SQC (30% more). Proteins from spots 1694 and 1697 were also regarded as potential isoforms and were also detected in higher abundance in N-SQC (25%). A similarity analysis conducted against proteins with the same functional annotation pointed toward mitochondria as their subcellular location. Proteins with this RNA recognition motif domain are described to play different roles including binding to heterogeneous nuclear ribonucleoproteins, small nuclear ribonucleoproteins, regulation of alternative splicing and regulation of RNA stability and translation. Polyadenylate-binding proteins are also RNA binding proteins, and one isoform was found to be more abundant in N-SQC (40%, spot 724). This protein was detected in four spots and all to be considered putative isoforms with no quantitative changes between the cork quality groups found.

One regulatory subunit of the 26S proteasome (superfamily IPR035263) was found to be differently abundant, showing a two-fold increase in N-SQC producing cells. This regulatory subunit is an ATPase with an AAA^+^ domain. The AAA^+^ superfamily of ATPases is involved in protein processing, transport and degradation^9^.

Two isoform candidates for ATP synthase were found to accumulate 30% more in N-SQC than in SQC. Additional 7 possible isoforms were identified as ATP synthase, and when all potential isoforms were considered in N-SQC producing cells, it exhibited 15% more of this protein. Once again, the similarity analysis pointed out for mitochondria as the most probable subcellular location for these isoforms candidates. The transport across the mitochondria outer membrane via porins also showed differences between the cork quality groups. Several porins were detected in our dataset, and a detailed analysis indicated the presence of the domain IPR027246. This feature supports a role as a translocator channel (Tom40) for unfolded protein across the outer mitochondrial membrane. One Tom40 isoform was found to be differently expressed (40% more abundant in N-SQC, spot 1220). The same trend was observed when all the ten putative isoforms were considered, with N-SQC producing cells exhibiting 25% more of this protein translocator.

Detailed peptide analysis allowed to confirm the identity of spots 1687 and 1709 as an acyl-carrier proteins (ACP), the volume of spot 1687 being 30% higher in SQC. Several other acyl-carrier-proteins (reductases and dehydrases) were identified. When the volume of the four acyl-carrier proteins was considered, no quantitative changes were found between the cork quality groups. These proteins function as cofactors in the *de novo* synthesis of fatty acids major constituents of suberin, cutin, and cellular membranes (Huang et al., 2017).

Stoppable quality cork also showed higher abundance of one Caffeoyl-CoA *O*-methyltransferase putative isoform (25% higher, spot 2856). But, when the volume of the five isoform candidates detected were considered, no quantitative changes could be found between the cork quality groups. Caffeoyl-CoA *O*-methyltransferase (EC 2.1.1.104) is a *S*-adenosyl-L-methionine-dependent enzyme (IPR029063) involved in the synthesis of different monolignols which are components of lignin (Zhong et al., 2000). Similarly to caffeoyl-CoA *O*-methyltransferase, no quantitative changes were found between the cork quality groups for *S*-adenosylmethionine synthetase (involved in cysteine and methionine metabolism as well as in the biosynthesis of secondary metabolites).

One presumed isoform, 2,3-bisphosphoglycerate-independent phosphoglycerate mutase, an enzyme involved in glycolysis, was assessed as differently accumulated in SQC (40 % higher volume, spot 513). But when considering the volume of all the three isoform candidates, no quantitative changes were found between the cork quality groups. Also, when all the glycolytic enzymes (and its several putative isoforms) were considered, no quantitative changes were found between the cork quality groups, the same trend was observed for the ROS metabolizing enzymes.

## Discussion

At the debarking, the proteome of the *Q. suber* cork producing cells showed high demand for energy and metabolic building blocks (carbon and amino acids; Figure 4), as previously observed by Ricardo et al. (2011). *Quercus* proteomes are largely under-represented in the literature (fewer than 25 papers by March 2013; Pinheiro et al., 2014) and to the best of our knowledge, only our study and that of Ricardo et al. (2011) have analyzed the cork producing proteome literature survey in December 2018 using the same sources and search criteria as Pinheiro et al. (2014).

The direct comparison of the proteome (this study) and the transcriptome (Teixeira et al., 2014) of cork producing cells showed differences between the two scales of analysis (Supplementary File S1 and Supplementary Figure S3), namely for the molecular function. The functional annotation of the proteome and transcriptome dataset showed a large preponderance of enzymes (catalytic activity) and binding. Proteome analysis, but not the transcriptome analysis, revealed high abundance of oxidoreductases (26%) and metal ion binding (16%) proteins The enzymes annotated under these functional categories included several dehydrogenases and enolases (involved in glycolysis and respiration) and signaling related proteins, such as annexins and other Ca^2+^ binding proteins.

Our study showed that proteome of recently formed suberized and un-suberized phellem, in combination with quali-quantitative analysis of soluble and cell wall-bound phenolics, can be used as proxy for the quality grade of the cork plank. Considering the relative costs of analysis, phenolics seems to provide a more accessible proxy of cork quality. Our study, showed that SQC producing cells made greater investment to the synthesis of cell wall-bound phenolics compared to N-SQC cells. It also revealed a large association of hydroxycinnamic acids and ferulate esters to cell wall polymers (via covalent bonds). The extent of ferulate oxidative cross-linking has been reported to influence the extensibility of cell wall and, consequently not only cell growth, but also pathogen accessibility (Mathew and Abraham, 2004). Consistently, our results, especially the content of coniferaldehyde, which represents a branch point of the biosynthesis of coniferyl and sinapyl alcohol (Pereira, 2007; Marques et al., 2016), was higher in SQC than in N-SQC, thereby indicating a more active lignin biosynthesis. On the contrary, N-SQC producing cells invested mostly in the biosynthesis of soluble phenolics, mainly hydrolysable tannins, consistently exhibiting a higher reducing capacity (more 40% compared to SQC producing cells; Figures 1, 2). Soluble phenolic compounds can play multiple cellular functions, from free radical scavenging (antioxidants) to the regulation of enzyme activity. Hydrolysable tannins (gallic acid esterified with sugars and forming oligomers or polymers) are effective antioxidants (Santos et al., 2010; Khoddami et al., 2013), with strong impact on ROS homeostasis and redox sensing, and hence, on the integration of external stimuli (Mhamdi and Van Breusegem, 2018; Waszczak et al., 2018). In addition, hydrolysable tannins have a role in the cross-linking and aggregation of proteins, contributing to biotic defense via anti-microbial activity (Dixon et al., 2002; Vogt, 2010). In our study, quantitative, but not qualitative differences were observed in both proteome and phenolic metabolism, therefore suggesting that the distinct cork quality is related to a fine metabolic pathway regulation. We speculate that cells integrating external stimuli to synthesize soluble hydrolysable-tannins at expenses of cell wall-bound phenolics, produce N-SQC, though the matter deserves further investigation.

The significance of phenolic metabolism on cork quality was further illustrated by group discrimination achieved when using protein, phenolic compounds or a combination of both (30% vs. 86% vs. 79%, respectively; Supplementary Figures S1C, S2D and Figure 5). The lower discrimination driven by proteomic analysis was likely due to low proteome polymorphism, since differences in the proteome were quantitative. Additionally, less than 10% of detected protein spots showed significant changes between quality groups. Our study revealed mitochondrial metabolism as one of the most distinctive feature of different quality groups, at the proteome level. Indeed, the abundance of several proteins with putative mitochondrial location was higher in N-SQC: these included ATP synthase, RNA-binding proteins, and unfolded protein translocation to the mitochondria, i.e., import of proteins synthetized *de novo* via Tom40, a membrane protein essential for import of protein precursors into mitochondria.

Albeit we have used dry weight as unit basis, equivalent number of cells couldn’t be assessed in the present work. In addition, cell wall thickness per cell also emerged as a relevant trait. It is, therefore, necessary to establish the correlation between cell division and elongation, as well as between cell wall composition and thickness. Microscopic evaluation of cell shape and dimension could provide useful information on G × E effects on cork growth. Cell number (phellogen activity) and cell height (cellular expansion) are under genetic control (Powell and Lenhard, 2012; Miguel et al., 2016) but are also strongly dependent on environmental conditions (Pereira, 2007). The local combination of temperature and soil water availability largely impacts cork growth (Costa et al., 2016), with differences in cork annual growth being observed between and within geographical regions (Lauw et al., 2018). The basis for such phenotypic plasticity relies on both cork oak genetic diversity and local adaptations (Elena-Rossellö and Cabrera, 1996; Coelho et al., 2006; Modesto et al., 2014; Eriksson, 2017; Lauw et al., 2018).

Our sampling occurred in two distinct geographical areas and, therefore, our data reflected G × E interactions. Trees in each “montado” originated from spontaneous sowing and most likely share the same genetic pool. As a consequence, the impact of environmental factors on both population fitness (evolutionary record) and individual performance (short-term effect) should influence the cork quality.

It is suggested that the analysis of recently formed phellogen from individuals grown in the same geographical area, with known cork quality production and genetic provenance, is crucial to solve the complex matter. This should allow to specifically disclose cork-quality related traits in the same environment and to further test the relative abundance of soluble hydrolysable tannins and cell-wall immobilized phenolics and how they related with cork-quality groups.

## Conclusion

Cork producing cells show a large preponderance of glycolysis, respiration and protein synthesis and degradation pathways, consistent with their meristematic nature. This observation is compatible with active cell growing phases, in which many proteins are produced and degraded according to the cell cycle phase. Active growth is further supported by a high respiratory demand. Redox regulation is also found relevant in cork producing cells, having the potential to regulate enzyme activity (e.g., glycolysis) and several other cell functions, including cell wall assembly and suberization. SQC and N-SQC producing cells exhibit similar protein patterns, suggesting fine regulations of the phellogen metabolic activity. One possible mechanism is through post-translational modification of the phellogen proteins, with impact on the meristematic activity, cell expansion and differential cell wall immobilization of phenolics. SQC producing cells exhibit a higher amount of cell wall-bound phenolics (hydroxycinnamic acids and hydroxybenzoic acids), which contrasts with N-SQC producing cells. N-SQC not only exhibits a higher amount of hydrolysable tannins but also differs in the type of most abundant component. Secondary metabolites confer a better hint to where we might find the actual key control mechanisms. In our study, distinct patterns of immobilized and soluble phenolics were observed concurrent with highly similar proteomes. We hypothesize that the observed differences in cork quality originates from: (i) regulation and activity of the enzymes involved in cell wall assembly and extracellular matrix dynamics, with strong impacts on cork thickness (ii) higher synthesis, transport and exocytosis of the cell wall components. The possibility to discriminate between SQC and N-SQC at molecular level provides a valuable diagnostic tool. Our study highlights hydrolysable tannins as promising metabolic markers. The validation of these metabolites would be very useful for the screening of individuals, especially if used early in selection.

## Data Availability

All datasets for this study are included in the manuscript and the Supplementary Files.

## Author Contributions

CP, CR, and RT designed the project. RT selected the trees and collected the samples. CP, MT, and CB supervised the protein and metabolic extraction as well as data analysis. SP and JR were responsible for the proteomic mass spectrometry. OZ, MT, CB, and AG performed the phenolic extraction and HPLC-MS/MS analysis. CP, SP, CB, JdA, and SW were involved in data curation, validation, and analysis. CP, CR, and RT were involved in funding acquisition. CP, CR, MT, JR, and SW provided the necessary resources. All authors contributed to the manuscript writing, and read and approved the final manuscript.

## Conflict of Interest Statement

The authors declare that the research was conducted in the absence of any commercial or financial relationships that could be construed as a potential conflict of interest.

## Footnotes

1 www.ipma.pt

2 http://phenol-explorer.eu

3 http://www.matrixscience.com

4 http://proteomecentral.proteomexchange.org

5 http://ezcatdb.cbrc.jp

6 http://www.ebi.ac.uk/interpro/entry/IPR008030/proteins-matched

7 http://www.ebi.ac.uk/interpro/entry/IPR002048

8 http://www.ebi.ac.uk/interpro/entry/IPR037104

9 http://www.ebi.ac.uk/interpro/entry/IPR003593

## Glossary

Phellem: aka as cork. Protective tissue composed of non-living suberized cells. It is produced by phellogen
Phelloid: unsuberized phellem
Phellogen: meristematic cell layers

## Abbreviations

N-SQC: non-stoppable quality cork
SQC: stoppable cork quality
