# Supplementary material for: Phellem Cell-Wall Components Are Discriminants of Cork Quality in Quercus suber
Source: Front Plant Sci. 2019 Jul 30;10:944. doi: 10.3389/fpls.2019.00944 (PMC6682605; doi:10.3389/fpls.2019.00944)
Supplement: Supplementary file 1 [file Table_1.docx]

Supplementary Table S1. HPLC-MS-MS characterization of phenolics of cork producing cells.

| Compounds | [M-H]^-^ *m/z* | MS^2^ ions (*m/z*) | λ_max_ (nm) | Literature Reference |
| --- | --- | --- | --- | --- |
| Monogalloyl-glucose | 331 | 271, 169 | 280 | Cantos *et al*. 2003 |
| Castalagin/Vescalagin | 933 | 631 | 247 | Fernandes *et al*. 2011 |
| Castalagin/Vescalagin derivative | 969 | 933, 631 | 247 | Fernandes *et al*. 2011 |
| Ellagic Acid | 301 | 229, 185 | 254, 368 | Fernandes *et al*. 2011 |
| Gallic Acid | 169 | 125 | 270 | Fernandes *et al*. 2011 |
| (+) Catechin | 289 | 245 | 280 | Callemien & Collin 2008 |
| Conyferaldeyde | 177 | 133, 121, 105 | 289, 340 | Fernandes *et al*. 2011 |
| Valoneic Acid dilactone | 469 | 425, 300 | 253, 373 | Fernandes *et al*. 2011 |

Cantos et al. 2003. *J.Agric.Food.Chem.* 51:6248-55;

Callemien & Collin 2008*. J.Am.Soc.Brew.Chem*. 66:109-115;

Fernandes et al. 2011*. J.Agric.FoodChem*. 57:11154-60
