# Supplementary material for: Phellem Cell-Wall Components Are Discriminants of Cork Quality in Quercus suber
Source: Front Plant Sci. 2019 Jul 30;10:944. doi: 10.3389/fpls.2019.00944 (PMC6682605; doi:10.3389/fpls.2019.00944)
Supplement: Supplementary file 2 [file Table_2.docx]

Supplementary Table S2. Caffeic acid derivatives immobilised in the cell walls of N-SQC and SCQ producing cells, detected by HPLC-MS/MS after base hydrolysis. Data show the means (μmol per g^-1^ sample dry weight) ± standard error (n=5). Significance levels between cork quality groups were assessed via Mann Whitney U test (** p<0.01).

|  | N-SQC | SQC | p-level |
| --- | --- | --- | --- |
| caffeic acid derivative | 5.42 ± 0.79 | 13.07 ± 0.52 | ** |
| caffeic acid derivative2 | 1.79 ± 0.38 | 2.92 ± 0.10 | ** |
| caffeic acid derivative3 | 0.54 ± 0.05 | 2.10 ± 0.11 | ** |
| caffeic acid derivative4 | 1.02 ± 0.16 | 2.98 ± 0.31 | ** |
| caffeic acid derivative5 | 1.78 ± 0.30 | 3.64 ± 0.32 | ** |
| caffeic acid derivative6 | 1.70 ± 0.21 | 3.78 ± 0.17 | ** |
