# Supplementary figures and images for: Phellem Cell-Wall Components Are Discriminants of Cork Quality in Quercus suber
Source: Front Plant Sci. 2019 Jul 30;10:944. doi: 10.3389/fpls.2019.00944 (PMC6682605; doi:10.3389/fpls.2019.00944)

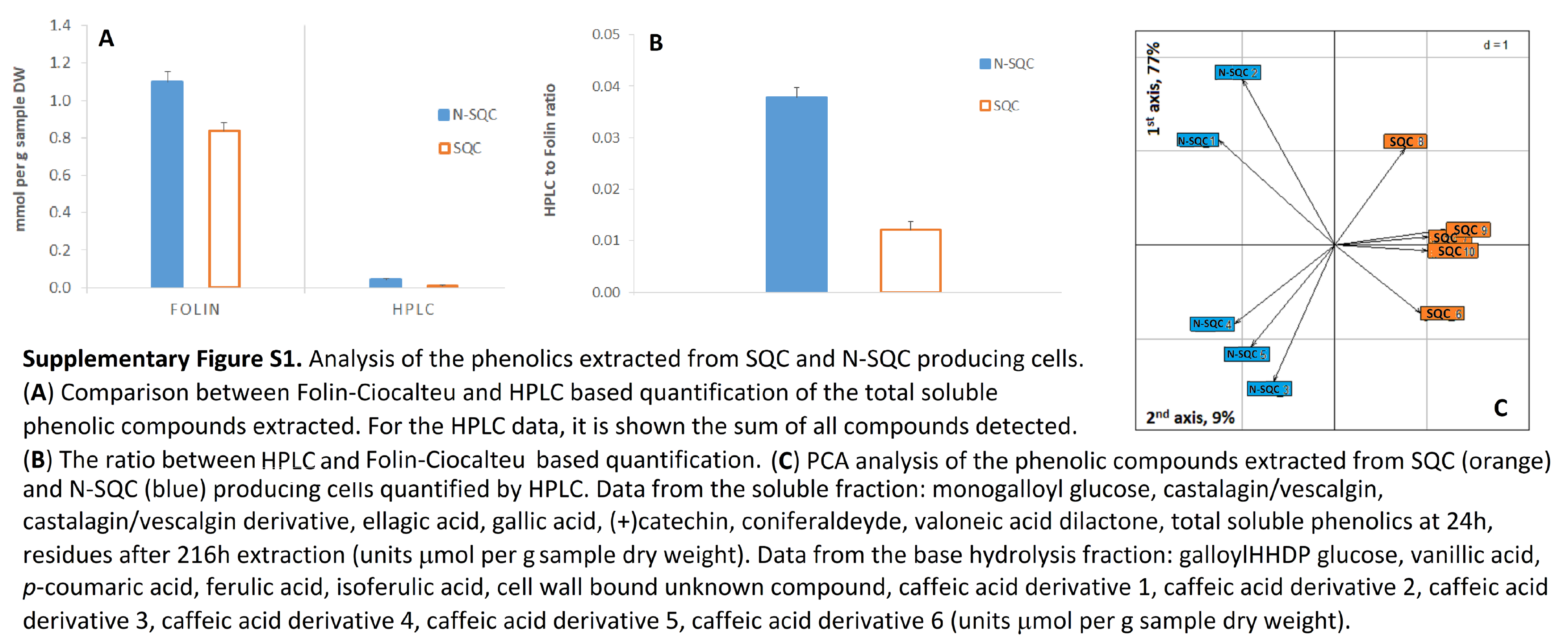

Supplement: Supplementary file 5 [file Image_1.TIF]

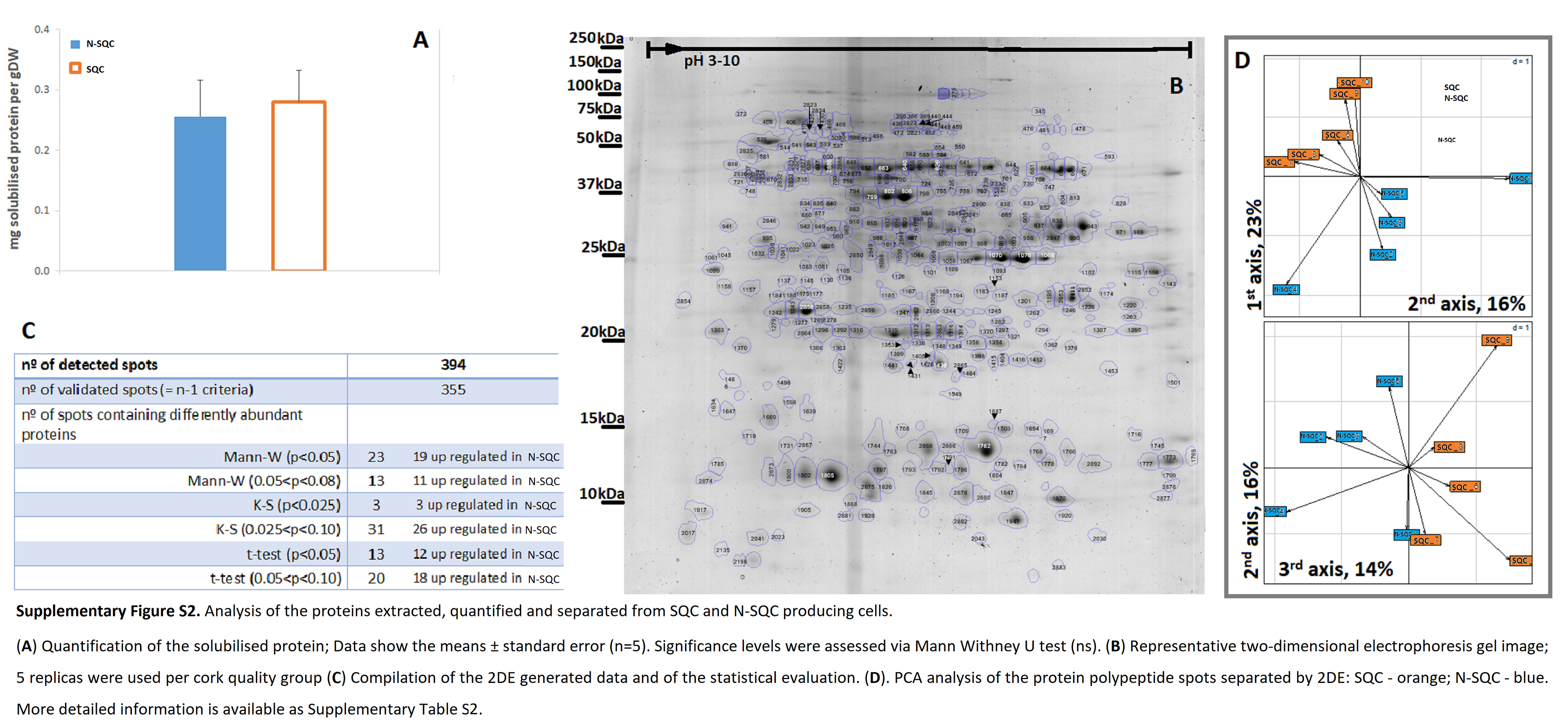

Supplement: Supplementary file 6 [file Image_2.TIF]
