# Supplementary material for: Phellem Cell-Wall Components Are Discriminants of Cork Quality in Quercus suber
Source: Front Plant Sci. 2019 Jul 30;10:944. doi: 10.3389/fpls.2019.00944 (PMC6682605; doi:10.3389/fpls.2019.00944)
Supplement: Supplementary file 7 [file Data_Sheet_1.docx]

**Supplementary File S1 – direct proteome and transcriptome comparison**

For a direct comparison of the proteome (this work) and the transcriptome already published (Teixeira *et al.*, 2014), the derived annotation for the whole set of proteins was preformed using the list of GO Slim nodes used in the transcriptome data set. Both datasets originate from the same sampling.

The derived annotation for the whole set of proteins was compared to a list of GO Slim nodes (the AGR set) from now on referred as categories. The match was made either against the actual nodes or their complete ontology trail. Whenever no match was available, the actual GO annotation for the protein was either retained or manually clustered to a meaningful higher order node. After separating the sets for each of the three base GO ontologies, each predefined category was assessed either for the number of protein spots annotated inside (Positive) or the remaining protein spots in that set. Due to a certain degree of natural overlapping both in the chosen categories, and multiplicity of activities annotated for some proteins, the total is bound to be equal or exceed the actual number of protein spots in the set. Every task in this process amenable to automation was carried out through Knime Platform ([www.knime.com/](http://www.knime.com/)) workflows. These were especially useful for automated WWW queries and parsing of the resulting documents. The location inside the cell was derived either from the InterPro GO annotation or from related protein similarity or phylogenetic analysis. Supplementary Figure S3 show the plotted results.

With this procedure, molecular function annotation was available for 78% of the proteins, while annotation for biological process and cellular component was lower (42 and 12 %, respectively). All the identified proteins are intracellularly located. The transcriptome data set also show a large preponderance of intracellular location of the gene products (95%) Considering the top 5 categories, the most represented biological process at the proteome level are carbohydrate metabolic process (26%), sulfur compound metabolic process (17%), response to stimulus (13%), protein metabolic process (11%), establishment of localization (10%). Carbohydrate and protein metabolic process were also among the top 5 categories in the transcriptome dataset, together with translation, lipid metabolism and stress response. Considering the molecular function, the functional annotation of the proteome and transcriptome dataset show a large preponderance of enzymes (catalytic activity) and binding. However, at the proteome level oxidoredutases (26%) and metal ion binding (16%) are shown to be highly represent and not appearing in the top 5 transcriptome categories.

**
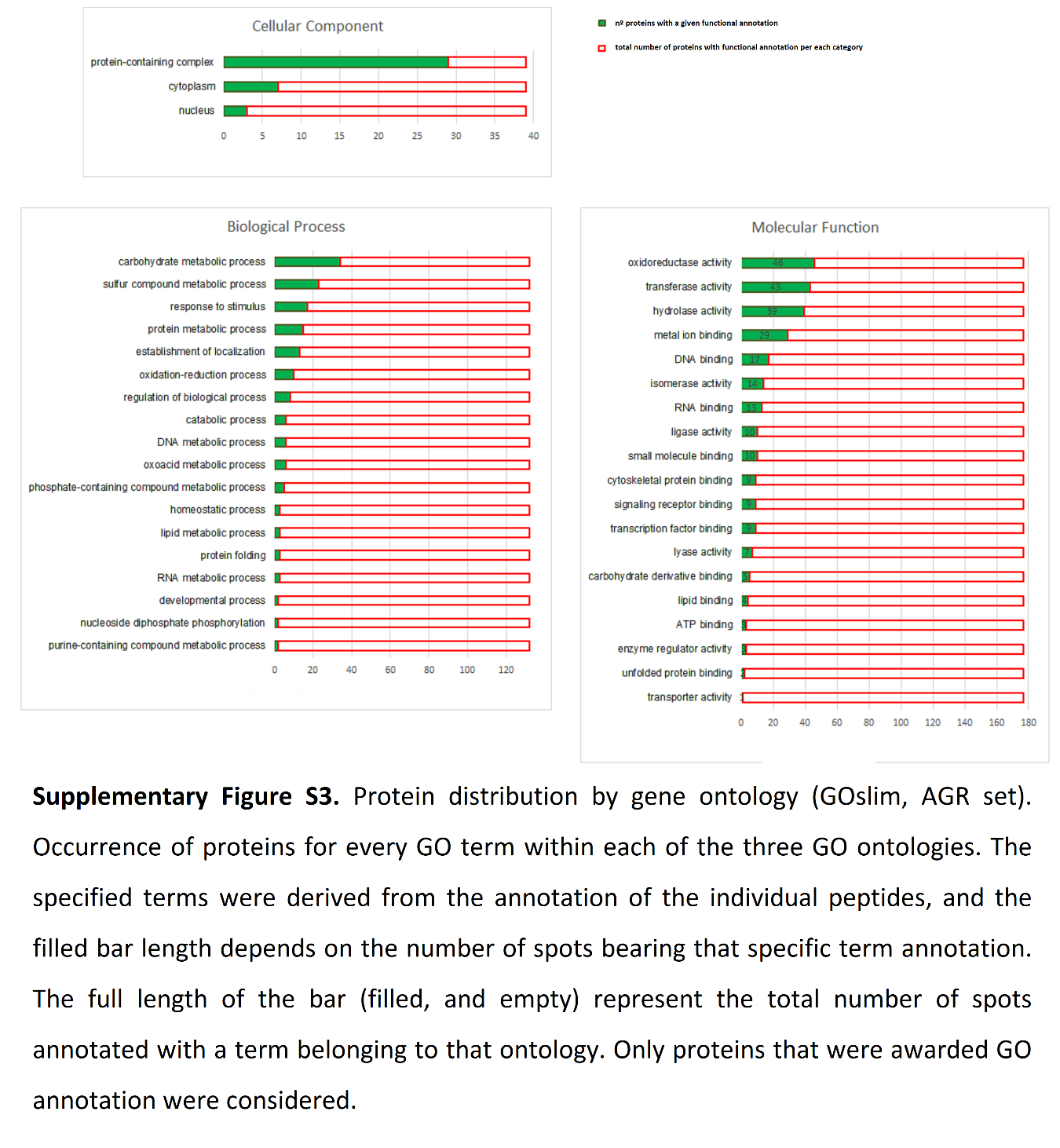
**

**Supplementary Figure S3.** Protein distribution by gene ontology (GOslim, AGR set). Occurrence of proteins for every GO term within each of the three GO ontologies. The specified terms were derived from the annotation of the individual peptides, and the filled bar length depends on the number of spots bearing that specific term annotation. The full length of the bar (filled, and empty) represent the total number of spots annotated with a term belonging to that ontology. Only proteins that were awarded GO annotation were considered.
